# Attenuation of Oxidative Damage via Upregulating Nrf2/HO-1 Signaling Pathway by Protease SH21 with Exerting Anti-Inflammatory and Anticancer Properties In Vitro

**DOI:** 10.3390/cells12172190

**Published:** 2023-09-01

**Authors:** Hasan Tarek, Seung Sik Cho, Md. Selim Hossain, Jin Cheol Yoo

**Affiliations:** 1 Department of Pharmacy, College of Pharmacy, Chosun University, Gwangju 61452, Republic of Korea; tarek@chosun.kr; 2 Department of Pharmacy, College of Pharmacy, Mokpo National University, Muan 58554, Republic of Korea; sscho@mokpo.ac.kr; 3 Department of Biomedicine, Health & Life Convergence Sciences, BK21 Four, Biomedical and Healthcare Research Institute, Mokpo National University, Muan 58554, Republic of Korea; 4 Department of Biomedical Sciences, Chosun University, Gwangju 61452, Republic of Korea; selim@chosun.kr

**Keywords:** protease SH21, oxidative damage, antioxidants, anti-inflammatory, anticancer

## Abstract

Oxidative damage and inflammation are among the very significant aspects interrelated with cancer and other degenerative diseases. In this study, we investigated the biological activities of a 25 kDa protease (SH21) that was purified from *Bacillus siamensis*. SH21 exhibited very powerful antioxidant and reactive oxygen species (ROS) generation inhibition activity in a dose-dependent approach. The mRNA and protein levels of antioxidant enzymes such as superoxide dismutase 1 (SOD1), catalase (CAT), and glutathione peroxidase 1 (GPx-1) were enhanced in the SH21-treated sample. SH21 also increased the transcriptional and translational activities of NF-E2-related factor 2 (Nrf2) with the subsequent development of detoxifying enzyme heme oxygenase-1 (HO-1). In addition, SH21 showed potential anti-inflammatory activity via inhibition of nitric oxide (NO) and proinflammatory cytokines, such as TNF-α, IL-6, and IL-1β, production in lipopolysaccharide (LPS)-stimulated RAW 264.7 cells. At concentrations of 60, 80, and 100 μg/mL, SH21 potentially suppressed nitric oxide synthase (iNOS) and cytokine gene expressions. Furthermore, SH21 significantly released lactate dehydrogenase (LDH) enzyme in cancer cell supernatant in a concentration-dependent manner and showed strong activity against three tested cancer cell lines, including HL-60, A549, and Hela. Our results suggest that SH21 has effective antioxidant, anti-inflammatory, and anticancer effects and could be an excellent therapeutic agent against inflammation-related diseases.

## 1. Introduction

Reactive oxygen species (ROS) can be formed when oxygen is partially reduced during normal metabolism. Damage to essential cellular components can result from oxidative stress occurring when antioxidant immunity is overloaded by ROS or loses its ability to respond [1]. It has been demonstrated that excessive ROS contributes to the etiology of a few human chronic illnesses involving inflammation, cardiovascular and neurological disorders, and cancer [2]. Depending on their mechanism, antioxidants can defend cells against oxidative stress, whether through direct or indirect pathways. Antioxidants remove reactive oxygen and nitrogen species by utilization or chemical transformation in the case of direct pathways. On the other hand, indirect pathways are involved in the upregulation of Phase II detoxifying and antioxidant enzymes. Aerobic microorganisms have robust antioxidant defense systems that include primary enzymes such as superoxide dismutase (SOD), catalase (CAT), glutathione peroxidase (GPx-1), and induced Phase II detoxifying enzymes such as heme oxygenase-1 (HO-1) and NAD(P)H quinone dehydrogenase 1 (NQO1), which are triggered by Nrf2 [3]. Many studies suggest that the Nrf2/HO-1 signaling pathways activate antioxidant enzymes that neutralize free radicals and prevent oxidative damage to cells. In normal conditions, the Keap1/Nrf2 complex, which is an adapter subunit within the cullin 3-based E3 ubiquitin ligase, controls the degradation of Nrf2 through the proteasome. Among many other things, the Nrf2/Keap1 pathway also plays a crucial role in regulating oxidative stress conditions in a way that contributes to inflammation, endothelial dysfunction, and cancer development [4]. The use of antioxidants has gained more attention for maintaining human health and the restraint and management of specific disorders. Synthetically produced antioxidants are commonly applied to treat oxidative damage. However, synthetic antioxidants have limited use due to their carcinogenicity, and the screening of natural biomaterial is currently an important consideration for several medical circumstances [5,6]. Thus, in controlling and healing some diseases, it might be beneficial to have an antioxidant molecule from environmental resources.

The acute inflammatory process is a biological consequence of vascularized active tissue in cases of bacterial infections and chemical or physical irritants. It contains the injury, eliminates invasive germs, inactivates poisons, and prepares the tissue or organ for recovery [7]. Generally, the development of inflammation is not regarded as an illness, but if it is not controlled and effectively resolved in a suitable manner, tissue damage and signaling pathways are modulated [8]. Chronic inflammation is implicated with a greater risk of tumor growth and cancer, as reported by experimental, medical, and epidemiological data, which have shown that it contributes to the onset of 15–20% of global malignancies [9]. Macrophages, which act as invisible cells, release a small amount of highly bioactive immune mediators, such as nitric oxide, that play a critical function in tissue healing and are involved in a variety of disorders, including atherosclerosis, inflammation, carcinogenesis, hypertension, obesity, and diabetes. Furthermore, ROS promotes the release of these mediators, increasing the presence of more macrophages in inflammatory sites, thereby spreading inflammation. Tumor invasion and metastasis are believed to be caused by persistent inflammation-induced cell recruitment, ROS release, and genetic instability. [10]. Cancer progression tends to be facilitated by oxidative stress and the inflammatory response [11]. Antioxidant and anti-inflammatory characteristics could be effective indicators for detecting anticancer agents. Therefore, experts have kept looking into screening new biological substances.

There has been extensive use of fermenting vegetables to obtain bioactive compounds. As a result of the high content of lactic acid-producing bacteria, kimchi is very beneficial for human health [12]. Koreans have been eating salted and fermented vegetables such as kimchi, which are an excellent source of micronutrients and adequate trace minerals, for more than 2000 years. Several reports stated that kimchi has antioxidant, anti-inflammatory, anticancer, antimicrobial, anti-atherosclerotic, anti-diabetic, and anti-obesity activities. In addition, the fermentation of microorganisms has long been a vital source of protease enzymes, which are widely accessible hydrolytic enzymes of physiological and economic importance in nature. Proteolytic microorganisms produce biologically active peptides from food resources, which are generally considered nutritious for their antioxidant properties. These enzymes are also used to treat certain diseases including necrosis, cancer, and cardiovascular syndrome. A few plant and microbial proteases have been identified and evaluated for their therapeutic potentials, including their antioxidant and anti-inflammatory properties [13,14,15,16]. The proteases which attracted attention due to their medicinal and biological applications are Bromelain, Capparin, Papanino, and Zingipain [17,18,19]. Previously, we reported the isolation and biochemical characterization of protease (SH21) from *Bacillus siamensis* [20], and as far as our knowledge, SH21 is the first protease with potential multifunctional biological activity which has been isolated from the Korean fermented food, kimchi.

In the present study, we evaluate antioxidant, anti-inflammatory and anticancer properties as evaluated by numerous in vitro tests. We also revealed the antioxidant mechanisms of SH21 by determining antioxidant enzyme expression and inducing HO-1 by the activation of Nrf2 in RAW 264.7 cells, and subsequently inhibited ROS generation and oxidative damage. These results suggest that SH21 reduces ROS free radicals and oxidation through the activation of the Nrf2/HO-1 pathway.

## 2. Materials and Methods

### 2.1. Materials

To perform experimental tests, all analytical grade reagents were employed. 2,2-diphenyl-1-picrylhydrazyl (DPPH), 2,2′-azino-bis(3-ethylbenzothiazoline-6-sulfonic acid (ABTS), Neocaprione, 2,4,6-Tris(2-pyridyl)-s-triazine (TPTZ), 3-(4,5-dimethylthiazol-2-yl)-2,5 diphenyltetrazolium bromide (MTT), PBS (phosphate-buffered saline, pH 7.4), Dimethylsulfoxide (DMSO), and DCFH-DA (2′,7′-Dichlorofluorescin diacetate) were procured from Sigma Aldrich (St. Louis, MO, USA). Dulbecco Modified Eagle Medium (DMEM), Roswell Park Memorial Institute (RPMI-1640), and fetal bovine serum were purchased from Gibco, Thermo Fisher Scientific (Waltham, MA, USA). Macrophage Raw 264.7 cells were collected from ATCC (Rockville, MD, USA). Lactate dehydrogenase (LDH) release assay kit was obtained from Cayman Chemical (Ann Arbor, MI, USA). Antibodies, including anti-Superoxide dismutase1 (SOD1), anti-Catalase (CAT), anti-Glutathione peroxidase 1 (GPx-1), anti-HO-1, anti-Nrf2, and β-actin and lamin B were obtained from Santa Cruz Biotechnology (Santa Cruz, CA, USA).

### 2.2. Cell Culture and Maintenance

All cells were cultured and maintained in a suitable medium. RAW 264.7 (Murine macrophage cell) and Hela cells (Human cervical cell) were grown in DMEM. HL-60 (Human leukemia cell) and A549 (Human lung carcinoma cell) cells were grown in RPMI-1640 medium combined with fetal bovine serum (FBS, 10%, *v*/*v*) and penicillin-streptomycin (100 μg/mL each) at 37 °C with 5% CO_2_. RAW 264.7 cells were obtained from ATCC (Rockville, MD, USA) and all three tested cancer cells were collected from Korean Cell Line Bank (KCLB, Seoul, Republic of Korea).

### 2.3. Production, Purification, and Molecular Weight Determination of SH21

Production and purification were carried out as described earlier [20]. Sodium dodecyl-sulfate polyacrylamide gel electrophoresis (SDS-PAGE) was performed to determine the molecular weight of SH21 according to Lamelli et al. [21]. After electrophoresis, the gel was stained with Coomassie Brilliant Blue R-250 to visualize the protein bands. PageRuler protein ladder (10–170 kDa) was used as a standard.

### 2.4. DPPH Radical Scavenging Activity Assay

The capacity to remove free radicals of SH21 was evaluated using the 2,2-diphenyl-1-picrylhydrazyl (DPPH) radical-scavenging assay, and the process was performed as reported earlier [22]. In brief, 190 μL of DPPH solution (0.2 mM, in 95% ethanol) was added to several concentrations (2.5–20 μg/mL) of 10 μL SH21. The mixture was then vortexed and kept for 30 min at normal temperature in a dark place. The resulting mixture absorbance was taken at 517 nm. Ascorbic acid was evaluated as a standard antioxidant. The percentage of inhibition was computed by applying the following equation.

$$\mathrm{DPPH}\;\mathrm{radical}\;\mathrm{scavenging}\;\mathrm{activity}\;(\%\;\mathrm{inhibition})=\left[\frac{\left(A_{control}-A_{sample}\right)}{A_{control}}\right]\times 100$$

Here, A_control_ and A_sample_ are the optical density of the control (blank) and sample, respectively. All samples were investigated three times.

### 2.5. ABTS Radical Scavenging Activity Assay

The 2,2′-azino-bis(3-ethylbenzothiazoline-6-sulfonic acid (ABTS) radical decolorization test was carried out following the method reported by Ye et al. [23]. The combination of 7 mM ABTS and 2.45 mM potassium persulfate (both in an equal volume of water) resulted in ABTS •+ cation radicals, which were then left in the dark for 16 h. Further dilution of the reaction mixture in methanol allowed for the measurement of an optical density of 0.706 ± 0.001 at 734 nm. The reaction between several concentrations of 10 μL SH21 and 190 μL of the ABTS •+ solution was allowed to proceed for 5 min, and the absorbance at 734 nm was taken. Ascorbic acid was evaluated as a standard antioxidant.

$$\mathrm{ABTS}\;•+\mathrm{radical}\;\mathrm{scavenging}\;\mathrm{activity}\;(\%\;\mathrm{inhibition})=\left[\frac{\left(A_{control}-A_{sample}\right)}{A_{control}}\right]\times 100$$

Here, A_control_ and A_sample_ are the optical density of the control (blank) and sample, respectively. All samples were investigated three times.

### 2.6. Superoxide Radical Scavenging Activity Assay

The test was conducted to examine the potential of SH21 to inhibit the development of formazan by scavenging the superoxide radicals (O_2_^−^) generated in the riboflavin–light–NBT system [24]. First, 1 mL of solution containing 0.1 mg NBT, 20 mg riboflavin, and EDTA (12 mM) in 50 mM sodium phosphate buffer (pH 7.6) was mixed with 0.5 mL of the sample. A fluorescence lamp was employed to illuminate the reaction mixture. The absorbance was measured at 590 nm soon after illumination. Gallic acid was utilized as a reference compound.

### 2.7. Hydroxyl Radical Scavenging Activity Assay

To investigate the ability of SH21 as a scavenger of hydroxyl radicals (•OH), a Fenton reaction was conducted using Fe^3+^ ascorbate-EDTA-H_2_O_2_ as the source of hydroxyl ions [25]. The reaction solution consists of 2.8 mM deoxyribose (400 μL) in KH_2_PO_4_-KOH buffer (pH 7.4), 0.1 M FeCl_3_ and 0.1 M EDTA (150 μL, 1:1 *v*/*v*), and 0.75 μL of 0.2 M H_2_O_2_ mixed with or without SH21. The reaction was started by mixing 0.75 μL of 0.3 M ascorbate and then left at 37 °C for 1 h. Then, 1 mL of TCA (2.8% *w*/*v*) was mixed with 0.5 mL of the reaction mixture before being combined with 1 mL of TBA (1% *w*/*v*). Finally, the resulting solution was incubated for 20 min at 100 °C. This test is based on the amount of the 2-deoxy-2-ribose sugar degradation product by condensation with 2-thiobarbituric acid (TBA) to generate a pink color. The color was observed at 532 nm.

### 2.8. Ferric Reducing Antioxidant Power (FRAP) Assay

A FRAP experiment was conducted to determine the reducing power activity of SH21 with minor modifications, as previously described [26]. The FRAP reagent consists of 2,4,6-Tris (2-pyridyl)-s-triazine (10 mM, TPTZ) mixture in HCL (40 mM), acetate buffer (300 mM, pH 3.5), and 20 mM ferric chloride (FeCl_3_·6H_2_O) solution in the following ratio: 8:88:8. Different concentrations of 10 μL SH21 and 190 μL of FRAP reagent were mixed and absorbance was taken at 595 nm. An ascorbic acid standard curve was employed to calculate the FRAP value.

### 2.9. Cupric Reducing Antioxidant Capacity (CUPRAC) Assay

The CUPRAC analysis was conducted by applying as reported before [27]. Several concentrations of SH21 were then mixed with a solution containing CuCl_2_ (10 mM), neocuproine (7.5 mM), and ammonium acetate buffer (1 M, pH 7.0). Absorbance measurement was performed at 450 nm after 1 h of incubation at normal temperature. Finally, the ascorbic acid standard curve was employed to estimate the CUPRAC value.

### 2.10. Cell Viability and Intracellular ROS Generation Inhibition in Raw 264.7 Cell

The viability of RAW 264.7 cells was assessed using the colorimetric (MTT) assay [28]. Prior to being treated with various concentrations (2.5 to 100 μg/mL) of SH21, RAW 264.7 cells were grown in 96-well plates at a density of 2 × 10^4^ cells/well for 24 h. The plates were incubated at 37 °C for 1 h afterward, and MTT reagent was added to each well after 24 h of incubation. After removing the medium, PBS (pH 7.4) was used to wash the plates twice. DMSO was utilized for dissolving the intracellularly insoluble formazan. The percentage (%) of cell viability was then calculated by taking absorbance at 570 nm with a microplate reader (Victor3, PerkinElmer, Waltham, MA, USA).

Cellular oxidative stress is caused by the reactive oxygen species (ROS) produced by LPS, which was quantified spectrofluorometrically employing the dichloro-dihydro-fluorescein diacetate (DCFH-DA) technique [28]. Raw 264.7 cells were first grown at a density of 2 × 10^5^ Raw 264.7 cells/well using DMEM in 96-well plates for 24 h. After treating them for 1 h with different concentrations (2.5 to 50 μg/mL) of SH21, cells were stimulated with LPS and kept for 24 h. Then, cells were treated with DCF-DA (25 μM) at 37 °C for half an hour after being cleansed twice with PBS (phosphate-buffered saline, pH 7.4). A fluorescent microplate reader was used to determine the fluorescence intensity at excitation and emission wavelengths of 485 nm and 528 nm, respectively.

### 2.11. Cell Lysates Preparation and Western Blot Analysis

RAW 264.7 cell lysates were made using a standard procedure, mixed with sample buffer consisting of Tris-HCl (250 mM, pH 6.8), 0.5 M dithiothreitol (DTT), glycerol (50%), bromophenol blue (0.5%,), SDS (10%), and 2-mercaptoethanol (5%), and denatured at 100 °C for 5 min. A nuclear/cytosolic fractionation kit (Cell Biolabs, Inc., San Diego, CA, USA) was employed for nuclear protein extraction. SDS-PAGE (10%) was employed to separate the sample proteins (20 μg), and the membranes were incubated overnight with the primary antibody in skim milk (5%, *w*/*v*) after electrotransfer on nitrocellulose membranes (Whatman, Dassel, Germany). Primary antibodies (1:1000), including anti-SOD1 (sc-101523), anti-CAT (sc-515782), anti-GPx-1 (sc-133152), anti-HO-1 (sc-136256), anti-Nrf2 (sc-81342), β-actin (sc-47778), and lamin B (sc-374015) (Santa Cruz Biotechnology, Inc., Santa Cruz, CA, USA), were applied. Anti-Mouse IgG-HRP (Santa Cruz) was employed as a secondary antibody. An ECL solution method was used to identify the antigen–antibody reaction. The density of the protein bands was normalized using the same samples on the β-actin.

### 2.12. Reverse Transcription Polymerase Chain Reaction (RT-PCR)

TRI-zol (Life Technologies, Gaithersburg, MD, USA) was applied to extract total RNA from Raw 264.7 cells according to the manufacturer’s protocols. Using an RT-&GO Mastermix (MP Biomedicals, Seoul, Republic of Korea), the RNA (2 μg) was transcribed into first-strand cDNA, and the resulting product was applied as the PCR template. The following primer (SOD1, CAT, GPx-1, HO-1, Nrf2, iNOS, TNF-α, IL-6, IL-1β, and GAPDH) sequences (Table 1) were employed in RT-PCR applying a Takara PCR thermal cycler. After electrophoresis, staining was performed with ethidium bromide to visualize the PCR results. Analyzing the bands was performed using Image Lab Software (version 5.2).

### 2.13. Nitric Oxide (NO) Generation Inhibition and Cytokines Assay

The Griess reaction was used to determine NO production by measuring the amounts of nitrite according to the method of Zhou et al. [29]. RAW 264.7 cells were seeded in 96-well plates at a density of 1 × 10^4^ cells/well. Before LPS stimulation, cells were pretreated with numerous concentrations (20 to 100 μg/mL) of SH21 for 2 h. After LPS (1 μg/mL) stimulation for 24 h, equal volumes (100 μL) of cultured supernatant and Griess reagent (1% sulfanilamide and 0.1% N-(1-Naphthyl) ethylenediamine dihydrochloride in 2.5% phosphoric acid) were added to 96-well plates and kept at room temperature for 10 min in a dark place. The absorbance was recorded at 540 nm using a microplate reader.

For the cytokines assay, RAW 264.7 cells were seeded in 48-well plates at a density of 1 × 10^4^ cells/well. Following attachment, cells were pretreated with various concentrations (20 to 100 μg/mL) of SH21 for 2 h and then stimulated for 24 h with LPS (1 μg/mL). The amounts of proinflammatory cytokines such as TNF-α (560478, BD Biosciences) IL-6 (550950, BD Biosciences), and IL-1β (569603, BD Biosciences) were measured in the supernatant by employing ELISA kits according to the manufacturer’s procedures. Absorbance was measured by using a microplate reader at 450 nm. All tests were performed in triplicate.

### 2.14. MTT Assay

The colorimetric MTT assay [30] was employed to determine the cytotoxicity of SH21 against three cancer cell lines (HL-60, A549, and Hela). 100 μL of cell suspension was seeded in 96-well microtiter plates at a density of 1 × 10^4^ cells/well and incubated at 37 °C for 24 h with 5% (*v*/*v*) CO_2_. Then, the medium in the wells was removed and various concentrations (50 to 500 μg/mL) of 100 μL SH21 solution were mixed and incubated for 24 h at 37 °C. Following incubation, the culture media was taken out and 50 μL of MTT dye solution (5 mg/mL) was applied to each well of the plate and incubated at 37 °C for 3 h. After withdrawing the MTT solution, 100 μL of DMSO was used to dissolve the formazan crystal. Finally, a microplate reader was used to determine the cell growth by taking absorbance at 540. Doxorubicin (0.25–10 μg/mL) was used as a positive control. The mean inhibitory concentration (IC_50_) was defined as the concentration that reduced cell viability by 50%. Each experiment was carried out three times.

### 2.15. The Lactate Dehydrogenase (LDH) Release Assay

The LDH leakage assay is a simple way of estimating and assessing lactate dehydrogenase enzyme release from lysed cancer cells. This assay was performed by using the LDH assay kit (Cat. No.: 601170). Briefly, all three cancer cells (HL-60, A549, and Hela) were plated in 96-well plates at a density of 5 × 10^3^ cells/well for 24 h and then incubated with different concentrations (50–500 μg/mL) of SH21 for 1 h. 100 µL of culture supernatant from each well was carefully transferred to new plates and then mixed with 100 µL of LDH reaction solution. Absorbance was measured at 490 nm after plates had been gently shaken at 37 °C for 30 min. Triton X-100 (1%, *v*/*v*) and phosphate buffer saline (PBS) in media were utilized as positive and negative controls, respectively. LDH release was expressed as a percentage compared to cells treated with Triton X-100. All experiments were performed three times.

### 2.16. Live/Dead Staining Assay

To investigate the cell membrane integrity, three cancer cells were seeded at a density of 3 × 10^4^ cells/well in 24-well plates and incubated for 24 h at 37 °C. After incubation, the culture medium was removed and again incubated with fresh medium containing SH21 at a concentration of 500 μg/mL for 24 h. Then, adhering cells were gently washed with PBS and cells were stained with 2 μM Calcein-AM and 5 μM Ehtidium homodimer-1 and placed in a dark place for 30 min. Finally, images were taken by using CLSM (Carl Zeiss LSM510 microscope, Jena, Germany).

### 2.17. Statistical Analysis

All tests were performed three times and the results were presented as mean (±) standard deviation. The statistical analysis was evaluated using the student’s *t*-test or a one-way ANOVA. Significant differences were defined as ** *p* < 0.01 and * *p* < 0.05.

## 3. Results and Discussion

### 3.1. Production, Purification, and Molecular Weight Determination of SH21

**Figure 1:**
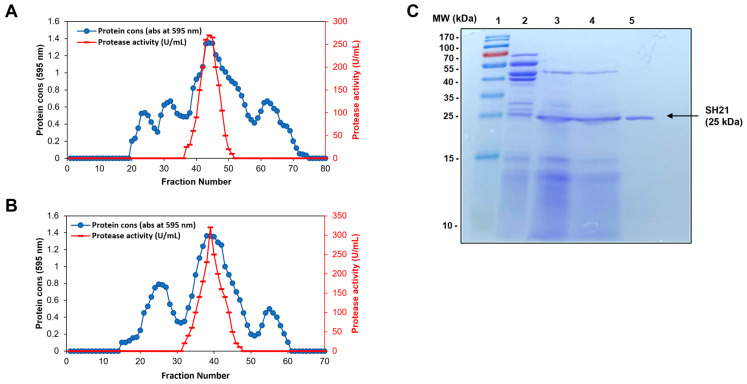
Purification procedure of SH21. (**A**) The elution profiles of Sepharose CL-6B (80 cm × 1.8 cm) and (**B**) Sephadex G-75 column (1.5 × 20 cm). (**C**) SDS-PAGE analysis of purified SH21. Lane 1, protein marker (10–170 kDa). Lane 2, crude sample Lane 3, ammonium sulfate precipitation (40–80%) Lane 4, sample after Sepharose CL-6B. Lane 5, purified SH21 after Sephadex G-75 column.

After the production of SH21, purification was carried out according to a three-step procedure including ammonium sulfate (40–80%), Sepharose CL-6B, and Sephadex G-75. Each purification step of SH21 is summarized in Table 2. The elution profiles of Sepharose CL-6B and Sephadex G-75 are shown in Figure 1. The purified SH21 was 22.25-fold pure with 19.23% yield and a specific activity of 2972.14 U/mg after final purification. To determine the molecular weight, SDS-PAGE was employed, and SH21 showed a single band of approximately 25 kDa, which suggests its homogeneity. The amino acid sequences and primary structure of SH21 were Q-T-G-G-S-F-F-E-P-F-N-S-Y-N-S-G-L-W-Q-K-A-N-G-Y-S [20]. The amino acid sequences of SH21 were rich in serine (S), glycine (G), phenylalanine (F), and asparagine (N). The amino acid position and length of a protein determine its biological activity [31]. It has also been reported that amino acids such as proline (P), alanine (A), phenylalanine (F), and leucine (L) contribute to antioxidant properties in proteins [32,33]. As a result of its low ionization potential, the pyrrolidine ring of proline can interact with the secondary structure of a protein to increase its flexibility and quench singlet oxygen. In the same way, one hydrogen atom from glycine (G) can contribute to the high flexibility of the peptide backbone as well as contribute to the antioxidant abilities of proteins. Additionally, glutamic acid (E) may contribute to antioxidant activity as well as act as a cation chelator [34].

### 3.2. Antioxidant Activity of SH21

Antioxidants provide protection against oxidation damage directly and indirectly to cells according to the mechanism that they use [35]. The capacity to scavenge free radicals, reactive oxygen, and nitrogen by donation of hydrogen or electrons may be characterized as direct antioxidant capacity. On the other hand, the indirect ability of antioxidants is involved in reducing oxidative stress through expressing phase II detoxifying and antioxidant genes. Investigation of whether SH21 possesses direct antioxidant activity relative to radicals of DPPH, ABTS •+, superoxide (O_2_^−^), and hydroxyl (•OH) was performed in terms of scavenging activities. To evaluate the capacity of compounds to act as free radical scavengers or hydrogen donors, comparatively stable DPPH radicals were extensively applied. The color intensity of the reaction mixture is proportional to the concentration and efficiency of antioxidants, as observed by the change in color from purple to yellow at 517 nm. A high level of free radicals leads to increased oxidation, which has a detrimental effect on biological systems [36]. The ABTS •+ radical scavenging assay generates blue/green chromophore radical cations released by the oxidation of ABTS •+ in the presence of potassium persulfate, which decline in the existence of hydrogen-contributing antioxidants.

**Figure 2:**
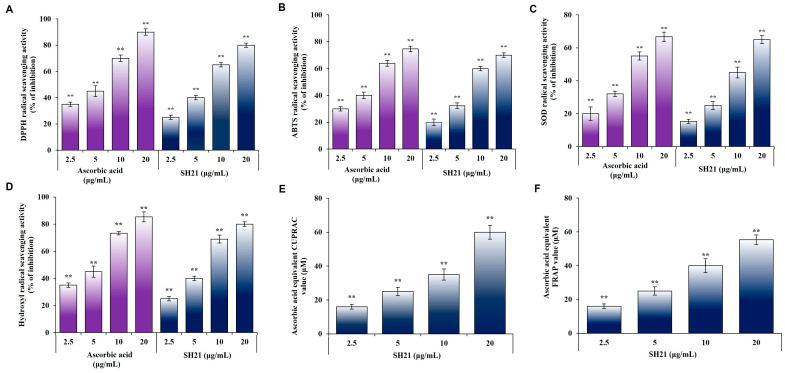
Antioxidant activity of protease SH21. The (**A**) DPPH assay, (**B**) ABTS assay, (**C**) Superoxide (SOD) radical scavenging assay, (**D**) Hydroxyl (HO) radical scavenging assay, (**E**) CUPRAC assay, and (**F**) FRAP assay were conducted with different concentrations of SH21, whereas ascorbic acid and gallic acid were used as standard antioxidant compounds. The reaction mixture without the sample was used as a negative control. All experiments were performed in triplicate. ** *p* < 0.01, significantly different from the control, using the student’s *t*-test.

Interestingly, SH21 displayed extreme scavenging activity for both DPPH and ABTS •+ radicals in a concentration-dependent approach (Figure 2A,B). Superoxide radical (O_2_^−^) is a highly toxic substance created by specific biological reactions. These radical anions, such as hydroxyl radicals and scavenging radicals, may play a role in the generation of highly reactive species even though they cannot initiate direct lipid oxidation, which is of great interest [37]. Hydroxyl radicals are highly reactive free radicals produced in the body and can damage almost any element that exists in living cells. Notably, SH21 substantially inhibited superoxide and hydroxyl radicals through the transfer of hydrogen atoms in a dose-dependent manner (Figure 2C,D). In addition, assays were carried out to evaluate the electron donating capacity of SH21 cupric-reducing antioxidant capacity (CUPRAC) and ferric-reducing antioxidant power (FRAP). SH21 displayed strong reducing power potentiality (Figure 2E,F). Based on these results, we predicted that SH21 has a high potential to scavenge different free radicals via hydrogen atom transfer/electron contribution.

### 3.3. Cell Cytotoxicity Effect and ROS Generation Inhibition of SH21

**Figure 3:**
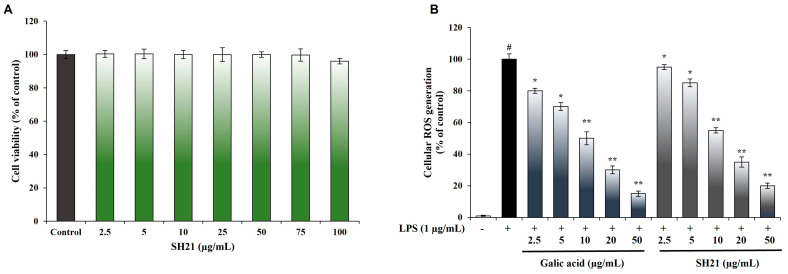
Investigation of cell viability and ROS generation inhibition. (**A**) RAW 264.7 cells were seeded at a density of 2 × 10^4^ cells per well and underwent an MTT assay. (**B**) Intracellular ROS generation. Each experiment was carried out three (n = 3) times (±) standard deviation. ^#^ *p* < 0.001, significantly different from normal control, * *p* < 0.05 and ** *p* < 0.01 significantly different from negative control, using the student’s *t*-test.

The toxic effects on RAW 264.7 cells with various concentrations (2.5 to 100 μg/mL) of SH21 were examined prior to investigating LPS-induced ROS scavenging activity. Reactive oxygen species (ROS) are dangerous to cellular function as they induce changes in nucleic acids, proteins, and lipids, ultimately leading to the development of inflammation, cancer, and neuron-related diseases [38]. The results showed that SH21 treatment inhibited ROS production in LPS-induced RAW 264.7 cells in a dose-dependent approach without showing cytotoxicity (Figure 3A,B).

### 3.4. Antioxidant Enzymes Expression by SH21 in Raw 264.7 Cells

**Figure 4:**
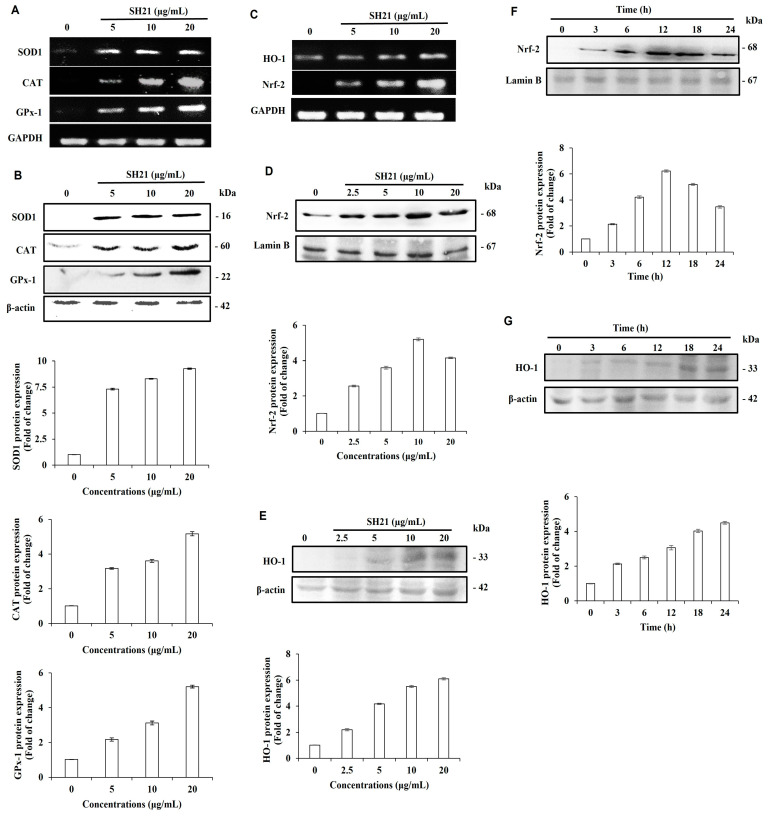
Evaluation of primary and phase II antioxidant and detoxifying enzymes. RAW 264.7 cells were pretreated for 24 h with various concentrations of SH21. (**A**) The mRNA expressions of the primary antioxidant enzyme and phase II antioxidant (SOD1, CAT, GPx-1) were measured by RT-PCR and (**B**) western blot was carried out to estimate protein levels by using the same concentrations. (**C**) The mRNA levels of detoxifying enzyme HO-1 and nuclear factor Nrf2 were measured by RT-PCR in a dose-dependent manner. The protein expressions of Nrf2 and HO-1 were measured in (**D**,**E**) dose-dependent and (**F**,**G**) time-dependent manners by western blot analysis. Each result presents the mean of three separate experiments (±) standard deviation.

The expression of antioxidant- and phase II-detoxifying enzymes protects against oxidative stress and facilitates ROS scavenging capability in sustaining cellular homeostasis during cell proliferation. Free radicals within cells are removed by enzymes, including SOD, CAT, and GPx-1. Damage to these enzymes is a contributing factor in some chronic diseases [39]. In general, one electron donation of oxygen (O_2_) on the mitochondrial electron transport chain is responsible for the production of cytosolic superoxide (O_2_^−^). It is widely acknowledged that SOD helps superoxide (O_2_^−^) convert into H_2_O_2._ In addition, other scavenging enzymes such as GPx-1 and CAT detoxified H_2_O_2_ into H_2_O. As part of the free radical metabolism pathway, all these enzymes work together [40]. To examine the mRNA expression and protein levels of antioxidant enzymes (SOD1, CAT, and GPx-1), SH21 was applied at concentrations of 5, 10, and 20 μg/mL to Raw 264.7 cells for 24 h and significantly increased the mRNA and protein levels in a dose-dependent manner (Figure 4A,B). In addition, RT-PCR analysis showed that the mRNA levels of phase II-detoxification enzyme (HO-1) and transcription factor Nrf2 prominently increased after SH21 treatment (Figure 4C).

Nrf2 (Nuclear factor erythroid 2-related factor 2) and Keap1 (Kelch-like ECH-associated protein) are usually linked in normal conditions, and the activation of phase II enzymes can only occur if Nrf2 is released from the Keap1 protein and translocated to the nucleus [41]. Furthermore, to examine the protein levels of HO-1 and Nrf2, western blot analysis was performed in both concentration and time-dependent (Figure 4D–G) approaches. SH21 increased protein expression of Nrf2, showing a maximum at 10 μg/mL at 12 h, whereas HO-1 peaked at 20 μg/mL at 24 h. Interestingly, protein levels of Nrf2 were slightly decreased after 12 h of treatment, but HO-1 protein levels were gradually improved until 24 h. HO-1 helps by changing heme into a strong pro-oxidant called biliverdin which has potential anti-inflammatory, antioxidant, and antiproliferative effects; it also converts to another powerful antioxidant called bilirubin [41,42]. Some nutrients such as caffeic acid ester, eckol, curcumin, and hydroxytyrosol have been found to help with oxidative stress by increasing HO-1 expression [43]. Thus, the mechanism of interaction between SH21 and Nrf2 is hypothesized to imitate the activity of other Nrf2 stimulants such as 5-O-caffoylquinic acid, which affects Nrf2 nuclear translocation and ARE-dependent gene expression as in GST, NQO-1, HO-1, in HT29 cells [44]. Based on this finding, SH21 indicates the upregulating of antioxidant enzymes by activating the Nrf2/HO-1 signaling pathway.

### 3.5. SH21 Exerts Anti-Inflammatory Activity

**Figure 5:**
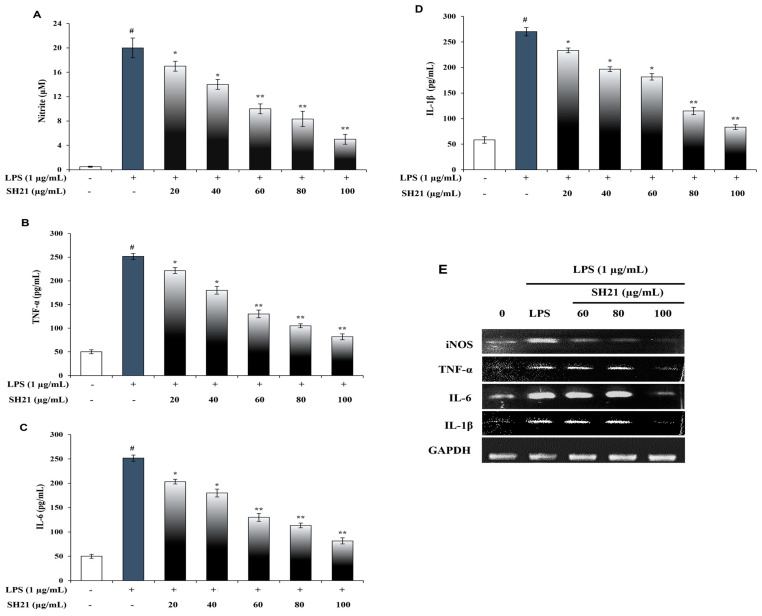
Anti-inflammatory activity of protease SH21. Effect of SH21 on the production of (**A**) NO, (**B**) TNF-α, (**C**) IL-6, and (**D**) IL-1β in LPS-induced RAW 264.7 cells. The cells were treated with SH21 at various concentrations of (20–100 µg/mL) for 2 h and then stimulated with LPS (1 µg/mL) for 24 h. Vertical bars indicate the mean (n = 3) ± standard deviation. (**E**) Effects of SH21 on LPS-induced mRNA expression of iNOS, TNF-α, IL-6 and IL-1β. Data expressed the means ± SD from three separate experiments. Statistically, significances were expressed as * *p* < 0.05 and ** *p* < 0.01. ^#^ *p* < 0.001, significantly different from normal control.

Macrophages are critical immune cells that can be activated by LPS (lipopolysaccharide). LPS is an endotoxin that exists in the cell surface of Gram-negative bacteria, which induces Raw 264.7 cells to produce mediators such as nitric oxide (NO) and proinflammatory cytokines (TNF-α, IL-6, IL-1β), leading to inflammation [45]. These cytokines may contribute to various pathophysiological conditions by further activating macrophage cells and stimulating the production of other inflammatory cytokines. The suppression of these cytokines is an important phase in the anti-inflammatory response [46]. The inhibitory effects of SH21 on chemical mediators and cytokines were checked to evaluate the anti-inflammatory function. In the presence of LPS, the levels of NO and cytokines were remarkably increased in Raw 264.7 cells that were analyzed by ELISA kits. SH21 showed significantly stronger inhibitory effects on the mediator and cytokines in a dose-dependent approach (Figure 5A–D). These results revealed that SH21 has potent anti-inflammatory activity. Furthermore, to evaluate whether the above inhibitory effect of SH21 on NO production and proinflammatory cytokines was related to changes in gene expressions, we examined the mRNA levels of iNOS, TNF-α, IL-6, and IL-1β by RT-PCR analysis. LPS treatment enhanced mRNA levels of iNOS, TNF-α, IL-6, and IL-1β, and treatment of cells with SH21 dose-dependently suppressed LPS-enhanced expression (Figure 5E). The attenuation of gene expressions indicates a positive effect of SH21 on anti-inflammatory properties.

### 3.6. Anticancer Activity of SH21

#### 3.6.1. Cell Viability and Cytotoxicity Assessment of SH21

**Figure 6:**
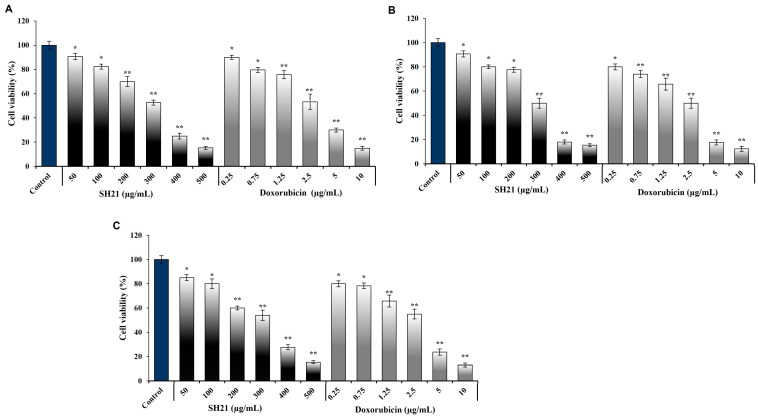
Anticancer activity of protease SH21 against three different cancer cell lines: (**A**) HL-60, (**B**) A549, and (**C**) Hela cells. Values were the means of triplicates (±) standard deviation (SD). Each experiment was perfomed three times (±) standard deviation. Significant differences were denoted as * *p* < 0.05 and ** *p* <0.01.

The anticancer effect of SH21 was assayed against three cancer cells, namely, HL-60, A549, and Hela cells. The anticancer activity was measured in terms of investigating cell viability and IC_50_ values. Cell growth was determined by MTT assay and was used to detect cells that were metabolically live. The three cancer cells were treated with various doses (50–500 μg/mL) of SH21. SH21 inhibited different percentages of cell growth in a dose-dependent manner (Figure 6A-C). Furthermore, IC_50_ values of 310.64 ± 0.24, 300.27 ± 45, and 317.14 ± 21 μg/mL against HL-60, A549, and Hela cells, respectively, were found after SH21 treatment. (Table 3). The lowest IC_50_ value of SH21 was observed against A549, whereas the highest IC_50_ value was against Hela cells. Doxorubicin (0.25–10 μg/mL) was used as a positive control and showed IC_50_ values of 2.4 ± 36, 2 ± 38, and 2.8 ± 74 μg/mL against HL-60, A549, and Hela cells separately (Table 3). The IC_50_ value refers to the concentration of the test sample that can inhibit the growth of cells by 50%. This result suggests that SH21 has considerable anticancer activity.

#### 3.6.2. Membrane Disruption Ability of SH21

**Figure 7:**
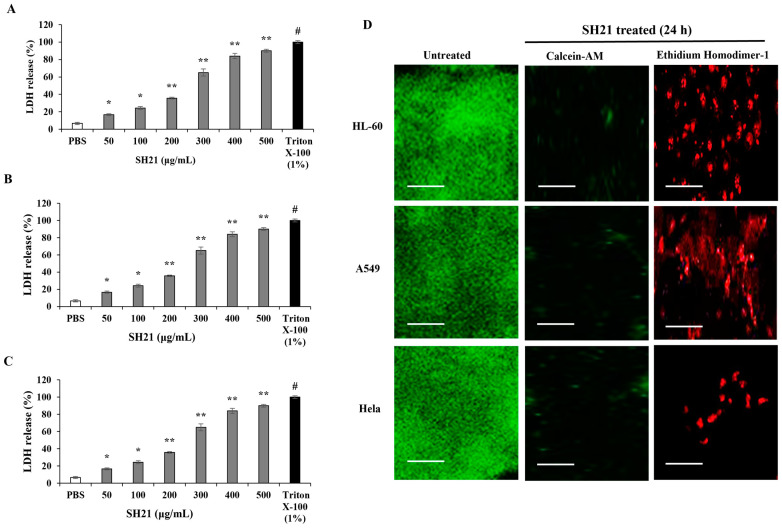
Membrane disruption ability of SH21 was assessed by observing LDH release and confocal microscopy assay. LDH leakage was monitored in three cancer cells, namely, (**A**) HL-60, (**B**) A549, and (**C**) Hela cells. All cancer cells were treated with different concentrations (50–500 μg/mL) of SH21, and absorbance were taken at 490 nm. PBS and Triton X-100 (1%, *v*/*v*) employed negative and positive controls, respectively. Values were the means of triplicates (±) standard deviation (SD). Significant differences were denoted as * *p* < 0.05 and ** *p* < 0.01. ^#^ *p* < 0.001. (**D**) Live/dead staining assay of SH21 against cancer cells (HL-60, A549, and Hela). Membrane damage was evaluated by employing confocal laser scanning microscopy (CLSM). All tested cancer cells were incubated with 500 μg/mL of SH21 for 24 h. Green fluorescence (Calcein-AM) and red fluorescence (Ethidium homodimer-1) indicated live and dead cells, respectively. Scale Bars = 100 µm.

Lactate dehydrogenase (LDH) is a stable cytoplasmic enzyme that is found in all cells, and it plays a role in the conversion of pyruvate to lactate. LDH is quickly released into the cell culture supernatant when the cell membrane is disrupted. The effect of SH21 on LDH release from tested cancer cells was investigated. Our result demonstrated that LDH was noticeably released from all tested cancer cells in a dose-dependent approach (Figure 7A–C), which indicates the disruption of the cell membranes.

In another experiment, we further verify the capability of SH21 to disrupt the cancer cell membrane. Calcein-AM, a cell-permeant nonfluorescent dye, is transformed to calcein by the esterase activity of living cells, suggesting an intact plasma membrane with green fluorescence. On the other hand, Ethidium homodimer-1 interacts with DNA and generates red signals when it penetrates the cell membrane. As shown in Figure 7, SH21 produced red fluorescence, which indicates damaged cell membranes in all the tested cancer cells. These results indicated SH21 has the potential ability to damage the cancer cell membrane.

**Figure 8:**
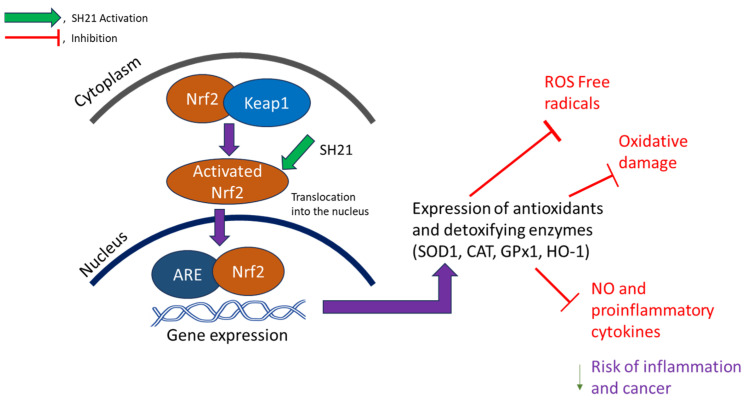
Hypothetical antioxidant mechanism of SH21.

Oxidation and reactive oxygen species (ROS) are key contributors to the cancer signature, including angiogenesis, invasion, stem cell function, and metastases. Therefore, it has been shown that reduction in oxidation and ROS free radicals with potent antioxidants would be an effective strategy for the prevention of cancer. While antioxidant compounds are implicated in numerous molecular processes, the possible antioxidant mechanism of SH21 may be regulated by nuclear translocation of Nrf2 into the nucleus, which activates HO-1 expression (Figure 8). This subsequently causes expression of antioxidant enzymes that inhibit ROS free radicals, oxidative damage, and proinflammatory cytokine production, which helps to decrease the chance of inflammation-related diseases and cancers. Since oxidative stress and inflammation play important roles in a variety of degenerative diseases, preventing them is one of the most effective ways to prevent disease. SH21 has been shown to maintain cellular homeostasis and protect cells from oxidative stress and inflammation by significantly increasing both the mRNA and protein levels of antioxidant enzymes such as SOD1, CAT, and GPx-1 in RAW264.7 cells and inhibiting ROS generation and proinflammatory cytokines. Therefore, it is interesting that a novel biological agent, such as SH21, can treat several oxidative stress-related pathophysiological conditions. In addition, our study demonstrated that SH21 has direct anti-inflammatory and anticancer properties. As far we know, no other antioxidative protease has ever been reported that reveals prominent anti-inflammatory and anticancer activity that was isolated and purified from the Korean fermented food, kimchi.

## 4. Conclusions

In the present study, SH21 revealed intense antioxidant activity, which was evaluated by various dose-dependent antioxidant assays. Additionally, SH21 increased the expression of antioxidants and detoxifying enzymes by activating the Nrf2/HO-1 pathway and prevented oxidative stress by inhibiting ROS generation in Raw 264.7 cells. In addition, SH21 exhibited substantial anti-inflammatory activity by downregulating NO and proinflammatory cytokines. Therefore, suppressing mRNA levels of iNOS and cytokines may help better our understanding of anti-inflammatory effectiveness. Subsequently, SH21 strongly inhibited the proliferation and disrupted the membrane of three tested cancer cells. Together, these results suggest that SH21 could be a potential therapeutic candidate for preventing oxidative damage and treating inflammation-related diseases. Upcoming studies are needed to prove the in vivo efficacy of SH21 in animal models.

## Figures and Tables

**Table 1:** List of primer sequences used in RT-PCR analysis.

Gene	Forward Primer Sequences (5′-3′)	Reverse Primer Sequences (5′-3′)
SOD1	AGG GCG TCA TTC ACT TCG AG	TCC TTT CCA GCA GCC ACA TT
CAT	AGG CTC AGC TGA CAC AGT TC	GCC ATT CAT GTG CCG ATG TC
GPx-1	GCT CAC CCG CTC TTT ACC TT	GAT GTC GAT GGT GCG AAA GC
HO-1	TGA GAG GAA CCA AGT GTT TGAG	CAG GGG GAC TTT AGC TTT AGAA
Nrf2	CTT TAG TCA GCG ACA GAA GGAC	TCC AGA GAG CTA TTG AGG GACT
iNOS	CCCTTCCGAAGTTTCTGGCAGCAGC	GGCTGTCAGAGCCTCGTGGCTTTGG
TNF-α	TCTCATCAGTTCTATGGCCC	GGGAGTAGACAAGGTACAAC
IL-6	GTTCTCTGGGAAATCGTGGA	TGTACTCCAGGTAGCTATGG
IL-1β	GGACGGACCCCAAAAGATG	AGAAGGTGCTCATGTCCTCA
GAPDH	GCG AGA TCC CGC TAA CAT CA	AGT GAT GGC ATG GAC TGT GG

**Table 2:** Purification summary of SH21.

Purification Steps	Total Protein (mg)	Total Activity (U)	Specific Activity (U/mg)	Purification Fold	Recovery (%)
Cell-free supernatant	324	43,272	133.55	1.00	100
Ammonium sulfate precipitation	166	31,254	188.28	1.41	72.22
Sepharose CL-6B	32	20,142	629.43	4.71	46.54
Sephadex G-75	2.4	8322	2972.14	22.25	19.23

**Table 3:** Cytotoxicity assessment of SH21 against three cancer cell lines.

SH21 (μg/mL)	Cell Type					
	HL-60		A549		Hela	
	% of Inhibition	IC_50_	% of Inhibition	IC_50_	% of Inhibition	IC_50_
50	9.33 ± 24	310.64 ± 24	9.31 ± 47	300.27 ± 45	15.15 ± 51	317.14 ± 21
100	17.67 ± 14		20.27 ± 51		20.51 ± 42	
200	30.14 ± 72		22.33 ± 57		40.38 ± 37	
300	47.33 ± 34		50.07 ± 42		46.36 ± 26	
400	75.28 ± 18		82.34 ± 63		72.33 ± 30	
500	84.67 ± 57		54.67 ± 17		84.67 ± 52	
**Doxorubicin (** **μg/mL)**	**% of** **inhibition**	**IC_50_**	**% of** **inhibition**	**IC_50_**	**% of** **inhibition**	**IC_50_**
0.25	10.13 ± 38	2.4 ± 36	20.32 ± 23	2.5 ± 38	20.18 ± 37	2.8 ± 74
0.75	20.21 ± 56		26.29 ± 29		21.67 ± 25	
1.25	24.33 ± 41		34.33 ± 83		34.33 ± 37	
2.50	46.67 ± 37		50.04 ± 25		45.34 ± 46	
5.0	70.15 ± 18		82.33 ± 54		76.33 ± 74	
10	85.17 ± 39		87.67 ± 32		87.61 ± 48	

Values are the means of triplicates ± standard deviation (SD).

## Data Availability

Data are available on request from the author.

## References

[1] Ames B.N., Shigenaga M.K., Hagen T.M. (1993). Oxidants, antioxidants, and the degenerative diseases of aging. Proc. Natl. Acad. Sci. USA.

[2] Belenky P., Collins J.J. (2011). Antioxidant strategies to tolerate antibiotics. Science.

[3] Kim J.-K., Jang H.-D. (2014). Nrf2-mediated HO-1 induction coupled with the ERK signaling pathway contributes to indirect antioxidant capacity of caffeic acid phenethyl ester in HepG2 cells. Int. J. Mol. Sci..

[4] Tossetta G., Fantone S., Marzioni D., Mazzucchelli R. (2023). Role of Natural and Synthetic Compounds in Modulating NRF2/KEAP1 Signaling Pathway in Prostate Cancer. Cancers.

[5] Saito M., Sakagami H., Fujisawa S. (2003). Cytotoxicity and apoptosis induction by butylated hydroxyanisole (BHA) and butylated hydroxytoluene (BHT). Anticancer Res..

[6] Botterweck A.A., Verhagen H., Goldbohm R.A., Kleinjans J., Van den Brandt P.A. (2000). Intake of butylated hydroxyanisole and butylated hydroxytoluene and stomach cancer risk: Results from analyses in the Netherlands cohort study. Food Chem. Toxicol..

[7] Philip M., Rowley D.A., Schreiber H. (2004). Inflammation as a tumor promoter in cancer induction. Semin. Cancer Biol..

[8] Serhan C.N., Savill J. (2005). Resolution of inflammation: The beginning programs the end. Nat. Immunol..

[9] Kuper H., Adami H.O., Trichopoulos D. (2001). Infections as a major preventable cause of human cancer. J. Intern. Med..

[10] Khan N., Afaq F., Mukhtar H. (2008). Cancer chemoprevention through dietary antioxidants: Progress and promise. Antioxid. Redox Signal..

[11] Arfin S., Jha N.K., Jha S.K., Kesari K.K., Ruokolainen J., Roychoudhury S., Rathi B., Kumar D. (2021). Oxidative stress in cancer cell metabolism. Antioxidants.

[12] Han H.-U., Lim C.-R., Park H.-K. (1990). Determination of microbial community as an indicator of kimchi fermentation. Korean J. Food Sci. Technol..

[13] Manivasagan P., Venkatesan J., Sivakumar K., Kim S.-K. (2013). Production, characterization and antioxidant potential of protease from *Streptomyces sp*. MAB18 using poultry wastes. BioMed Res. Int..

[14] Sangeetha R., Arulpandi I. (2019). Anti-inflammatory activity of a serine protease produced from *Bacillus pumilus* SG2. Biocatal. Agric. Biotechnol..

[15] Chanalia P., Gandhi D., Jodha D., Singh J. (2011). Applications of microbial proteases in pharmaceutical industry: An overview. Rev. Res. Med. Microbiol..

[16] Hellgren L., Mohr V., Vincent J. (1986). Proteases of Antarctic krill—A new system for effective enzymatic debridement of necrotic ulcerations. Experientia.

[17] Morris J.S., Friston K.J., Büchel C., Frith C.D., Young A.W., Calder A.J., Dolan R.J. (1998). A neuromodulatory role for the human amygdala in processing emotional facial expressions. Brain.

[18] Foy C., Passmore A., Vahidassr M., Young I., Lawson J. (1999). Plasma chain-breaking antioxidants in Alzheimer’s disease, vascular dementia and Parkinson’s disease. Qjm.

[19] Fusco D., Colloca G., Monaco M.R.L., Cesari M. (2007). Effects of antioxidant supplementation on the aging process. Clin. Interv. Aging.

[20] Tarek H., Nam K.B., Kim Y.K., Suchi S.A., Yoo J.C. (2023). Biochemical Characterization and Application of a Detergent Stable, Antimicrobial and Antibiofilm Potential Protease from *Bacillus siamensis*. Int. J. Mol. Sci..

[21] Laemmli U.K. (1970). Cleavage of structural proteins during the assembly of the head of bacteriophage T4. Nature.

[22] Khan M.M., Kim Y.K., Bilkis T., Suh J.-W., Lee D.Y., Yoo J.C. (2020). Reduction of oxidative stress through activating the Nrf2 mediated HO-1 antioxidant efficacy signaling pathway by MS15, an antimicrobial peptide from *Bacillus velezensis*. Antioxidants.

[23] Ye N., Hu P., Xu S., Chen M., Wang S., Hong J., Chen T., Cai T. (2018). Preparation and characterization of antioxidant peptides from carrot seed protein. J. Food Qual..

[24] Kumaran A., Karunakaran R.J. (2007). In vitro antioxidant activities of methanol extracts of five *Phyllanthus* species from India. LWT-Food Sci. Technol..

[25] Saeed N., Khan M.R., Shabbir M. (2012). Antioxidant activity, total phenolic and total flavonoid contents of whole plant extracts *Torilis leptophylla* L.. BMC Complement. Altern. Med..

[26] Benzie I.F., Strain J.J. (1996). The ferric reducing ability of plasma (FRAP) as a measure of “antioxidant power”: The FRAP assay. Anal. Biochem..

[27] Apak R., Güçlü K., Özyürek M., Karademir S.E. (2004). Novel total antioxidant capacity index for dietary polyphenols and vitamins C and E, using their cupric ion reducing capability in the presence of neocuproine: CUPRAC method. J. Agric. Food Chem..

[28] Rahman M.S., Choi Y.H., Choi Y.S., Alam M.B., Lee S.H., Yoo J.C. (2018). A novel antioxidant peptide, purified from *Bacillus amyloliquefaciens*, showed strong antioxidant potential via Nrf-2 mediated heme oxygenase-1 expression. Food Chem..

[29] Zhou H.Y., Shin E.M., Guo L.Y., Youn U.J., Bae K., Kang S.S., Zou L.B., Kim Y.S. (2008). Anti-inflammatory activity of 4-methoxyhonokiol is a function of the inhibition of iNOS and COX-2 expression in RAW 264.7 macrophages via NF-κB, JNK and p38 MAPK inactivation. Eur. J. Pharmacol..

[30] Chen Z., Xi X., Lu Y., Hu H., Dong Z., Ma C., Wang L., Zhou M., Chen T., Du S. (2021). In vitro activities of a novel antimicrobial peptide isolated from *phyllomedusa tomopterna*. Microb. Pathog..

[31] Morgan P.E., Pattison D.I., Davies M.J. (2012). Quantification of hydroxyl radical-derived oxidation products in peptides containing glycine, alanine, valine, and proline. Free Radic. Biol. Med..

[32] Joondan N., Laulloo S.J., Caumul P., Kharkar P.S. (2019). Antioxidant, antidiabetic and anticancer activities of L-Phenylalanine and L-Tyrosine ester surfactants: In vitro and in silico studies of their interactions with macromolecules as plausible mode of action for their biological properties. Curr. Bioact. Compd..

[33] Li Y., Jiang B., Zhang T., Mu W., Liu J. (2008). Antioxidant and free radical-scavenging activities of chickpea protein hydrolysate (CPH). Food Chem..

[34] Díaz M., Dunn C.M., McClements D.J., Decker E.A. (2003). Use of caseinophosphopeptides as natural antioxidants in oil-in-water emulsions. J. Agric. Food Chem..

[35] Dinkova-Kostova A.T., Talalay P. (2008). Direct and indirect antioxidant properties of inducers of cytoprotective proteins. Mol. Nutr. Food Res..

[36] Jamir K., Seshagirirao K. (2018). Purification, biochemical characterization and antioxidant property of ZCPG, a cysteine protease from *Zingiber montanum* rhizome. Int. J. Biol. Macromol..

[37] Kanatt S.R., Chander R., Sharma A. (2007). Antioxidant potential of mint (*Mentha spicata* L.) in radiation-processed lamb meat. Food Chem..

[38] Gibanananda R., Sayed A. (2002). Oxidants, antioxidants and carcinogenesis. Indian J. Exp. Biol.

[39] Srivastava S., Sinha D., Saha P., Marthala H., D’silva P. (2014). Magmas functions as a ROS regulator and provides cytoprotection against oxidative stress-mediated damages. Cell Death Dis..

[40] Wu J.Q., Kosten T.R., Zhang X.Y. (2013). Free radicals, antioxidant defense systems, and schizophrenia. Prog. Neuro-Psychopharmacol. Biol. Psychiatry.

[41] Zou X., Gao J., Zheng Y., Wang X., Chen C., Cao K., Xu J., Li Y., Lu W., Liu J. (2014). Zeaxanthin induces Nrf2-mediated phase II enzymes in protection of cell death. Cell Death Dis..

[42] Jin C.H., Lee H.J., Park Y.D., Choi D.S., Kim D.S., Kang S.-Y., Seo K.-I., Jeong I.Y. (2010). Isoegomaketone inhibits lipopolysaccharide-induced nitric oxide production in RAW 264.7 macrophages through the heme oxygenase-1 induction and inhibition of the interferon-β-STAT-1 pathway. J. Agric. Food Chem..

[43] Dinkova-Kostova A.T., Wang X.J. (2011). Induction of the Keap1/Nrf2/ARE pathway by oxidizable diphenols. Chem.-Biol. Interact..

[44] Boettler U., Sommerfeld K., Volz N., Pahlke G., Teller N., Somoza V., Lang R., Hofmann T., Marko D. (2011). Coffee constituents as modulators of Nrf2 nuclear translocation and ARE (EpRE)-dependent gene expression. J. Nutr. Biochem..

[45] Medzhitov R. (2010). Inflammation 2010: New adventures of an old flame. Cell.

[46] Chawla A., Nguyen K.D., Goh Y.S. (2011). Macrophage-mediated inflammation in metabolic disease. Nat. Rev. Immunol..

